# Supplementary material for: Crawling and Gliding: A Computational Model for Shape-Driven Cell Migration
Source: PLoS Comput Biol. 2015 Oct 21;11(10):e1004280. doi: 10.1371/journal.pcbi.1004280 (PMC4619082; doi:10.1371/journal.pcbi.1004280)
Supplement: S1 Code — (ZIP) [file pcbi.1004280.s012.zip › release/tst/doc/html/classParameter-members.html]

Tissue Simulation Toolkit: Member List


Tissue Simulation Toolkit
 0.1.4.1


- Main Page
- Namespaces
- Classes
- Files

- Class List
- Class Hierarchy
- Class Members

Parameter Member List

This is the complete list of members for Parameter, including all inherited members.

|  |  |  |
| --- | --- | --- |
| border\_energy | Parameter |  |
| chemotaxis | Parameter |  |
| CleanUp(void) | Parameter |  |
| conn\_diss | Parameter |  |
| datadir | Parameter |  |
| decay\_rate | Parameter |  |
| diff\_coeff | Parameter |  |
| divisions | Parameter |  |
| dt | Parameter |  |
| dx | Parameter |  |
| extensiononly | Parameter |  |
| graphics | Parameter |  |
| Jtable | Parameter |  |
| lambda | Parameter |  |
| lambda2 | Parameter |  |
| mcs | Parameter |  |
| n\_chem | Parameter |  |
| n\_init\_cells | Parameter |  |
| neighbours | Parameter |  |
| Parameter() | Parameter |  |
| pde\_its | Parameter |  |
| periodic\_boundaries | Parameter |  |
| Read(const char \*filename) | Parameter |  |
| relaxation | Parameter |  |
| rseed | Parameter |  |
| saturation | Parameter |  |
| secr\_rate | Parameter |  |
| size\_init\_cells | Parameter |  |
| sizex | Parameter |  |
| sizey | Parameter |  |
| storage\_stride | Parameter |  |
| store | Parameter |  |
| subfield | Parameter |  |
| T | Parameter |  |
| target\_area | Parameter |  |
| target\_length | Parameter |  |
| vecadherinknockout | Parameter |  |
| Write(ostream &os) const | Parameter |  |
| ~Parameter() | Parameter |  |


---

Generated on Thu Aug 14 2014 22:04:01 for Tissue Simulation Toolkit by  

 1.8.6
